# Common and Distinct Disruptions of Cortical Surface Morphology Between Autism Spectrum Disorder Children With and Without *SHANK3* Deficiency

**DOI:** 10.3389/fnins.2021.751364

**Published:** 2021-10-28

**Authors:** Dongyun Li, Chunxue Liu, Ziyi Huang, Huiping Li, Qiong Xu, Bingrui Zhou, Chunchun Hu, Ying Zhang, Yi Wang, Jingxin Nie, Zhongwei Qiao, Dazhi Yin, Xiu Xu

**Affiliations:** ^1^Department of Child Health Care, Children’s Hospital of Fudan University, Shanghai, China; ^2^Shanghai Key Laboratory of Brain Functional Genomics (Ministry of Education), School of Psychology and Cognitive Science, Affiliated Mental Health Center, East China Normal University, Shanghai, China; ^3^School of Psychology, South China Normal University, Guangzhou, China; ^4^Department of Radiology, Children’s Hospital of Fudan University, Shanghai, China

**Keywords:** surface-based morphometry, structural covariance networks, autism spectrum disorder, *SHANK3*, children

## Abstract

SH3 and Multiple Ankyrin Repeat Domains 3 (*SHANK3*)-caused autism spectrum disorder (ASD) may present a unique opportunity to clarify the heterogeneous neuropathological mechanisms of ASD. However, the specificity and commonality of disrupted large-scale brain organization in *SHANK3-*deficient children remain largely unknown. The present study combined genetic tests, neurobehavioral evaluations, and magnetic resonance imaging, aiming to explore the disruptions of both local and networked cortical structural organization in ASD children with and without *SHANK3* deficiency. Multiple surface morphological parameters such as cortical thickness (CT) and sulcus depth were estimated, and the graph theory was adopted to characterize the topological properties of structural covariance networks (SCNs). Finally, a correlation analysis between the alterations in brain morphological features and the neurobehavioral evaluations was performed. Compared with typically developed children, increased CT and reduced nodal degree were found in both ASD children with and without *SHANK3* defects mainly in the lateral temporal cortex, prefrontal cortex (PFC), temporo-parietal junction (TPJ), superior temporal gyrus (STG), and limbic/paralimbic regions. Besides commonality, our findings showed some distinct abnormalities in ASD children with *SHANK3* defects compared to those without. Locally, more changes in the STG and orbitofrontal cortex were exhibited in ASD children with *SHANK3* defects, while more changes in the TPJ and inferior parietal lobe (IPL) in those without *SHANK3* defects were observed. For the SCNs, a trend toward regular network topology was observed in ASD children with *SHANK3* defects, but not in those without. In addition, ASD children with *SHANK3* defects showed more alterations of nodal degrees in the anterior and posterior cingulate cortices and right insular, while there were more disruptions in the sensorimotor areas and the left insular and dorsomedial PFC in ASD without *SHANK3* defects. Our findings indicate dissociable disruptions of local and networked brain morphological features in ASD children with and without *SHANK3* deficiency. Moreover, this monogenic study may provide a valuable path for parsing the heterogeneity of brain disturbances in ASD.

## Introduction

SH3 and Multiple Ankyrin Repeat Domains 3 (*SHANK3*) is a master postsynaptic density scaffolding protein that is crucial for synaptogenesis, dendritic spine maturation, and synapse formation (Jiang and Ehlers, 2013; Monteiro and Feng, 2017). Mutations in genes coding for synaptic proteins consist of the most well-characterized genetic deficits reported in autism spectrum disorder (ASD; de la Torre-Ubieta et al., 2016). Haploinsufficiency of the *SHANK3* gene is a key contributor of autistic features in the 22q13.3 deletion syndrome (i.e., Phelan–McDermid syndrome; Phelan, 2008; Bonaglia et al., 2011; Phelan and McDermid, 2012). Supported by the fact of an approximately 2% prevalence in ASD patients with cognitive deficits, the *SHANK3* gene is considered as one of the most common genetic causes for ASDs (Betancur and Buxbaum, 2013; Leblond et al., 2014).

Magnetic resonance imaging (MRI), as a noninvasive examination tool, has been extensively applied to human ASD to delineate the brain morphometry and connectivity (Hazlett et al., 2005; Muller and Fishman, 2018; van Rooij et al., 2018). Numerous neuroimaging data in literature indicated alterations in brain morphology [e.g., cortical surface area, cortical thickness (CT), and gray matter volume] mainly in the frontal cortex, temporal cortex, and limbic/paralimbic regions (Hazlett et al., 2005; Ecker et al., 2015; Li et al., 2017). However, the majority of previous findings are inconsistent. The main reason could be the etiological and clinical heterogeneity of ASD. To address these caveats, one of the promising strategies is to conduct the study in a homogenous subgroup of ASD with confirmed genetic etiology. *SHANK3*-caused ASD thus presents a unique opportunity to understand the underlying neuropathological mechanisms.

Previous MRI studies in *SHANK*3-deficit patients are mostly single cases or case series with unsystematic approaches by visual MRI inspection resulting in a variety of gray and white matter structures found to be abnormal (Philippe et al., 2008; Soorya et al., 2013; Srivastava et al., 2019). A latest research from our team applying automated and unbiased whole-brain analyses to a relatively large sample revealed changes of striatum-centered gray matter volume in *SHANK3-*deficient children, suggesting possible alterations in large-scale brain networks (Liu et al., 2021). Although the volumetric morphometry is widely used to assess the brain structural alterations, it may still present some limitations that can be surpassed with surface-based morphometry (SBM). The cerebral cortex is arranged in a highly folded sheet with the majority of the cortical surface area buried in folds (Fischl et al., 1999). Surface-based algorithms can account for a better dissociation of information content and new forms of analyses regarding measuring cortical complexity in 3D space (Ghosh et al., 2010; Oosterhof et al., 2011). The surface measures such as gyrification index (GI) and sulcus depth (SD) allow the characterization of the cortical complexity, implicating the association with neurodevelopmental disorders, schizophrenia, and other cognitive disorders including ASD (Van Essen et al., 2006; Yang et al., 2016; Tang et al., 2021). However, little is known so far about the features of cortical surface morphology in patients with *SHANK3* deficit.

Beyond local morphological analysis, graph theoretical approaches to structural covariance networks (SCNs) offer a useful way to characterize the topological organization of the brain structure. The basic assumption underlying SCNs is that morphological correlations are related to axonal connectivity between brain regions with shared trophic, genetic, and neurodevelopmental influences (Alexander-Bloch et al., 2013b; Seidlitz et al., 2018). Researchers recently began to study the associations between the SCNs and the intrinsic functional networks for salience, executive function, and default mode in both neurotypical children and adults (Zielinski et al., 2010; Bruno et al., 2017). In individuals with ASD, previous investigations of SCNs have reported a decrease in various regional or nodal topological properties mainly in the regions of insula, hippocampus, caudate, prefrontal cortex (PFC), and the regions related to social and sensorimotor processing as well as speech and language (Vissers et al., 2012; Sharda et al., 2016). To date, however, no study has uncovered how the topological architecture of SCNs disrupted in *SHANK3-*deficient ASD. This approach could help to further elucidate the role of dysconnectivity in both of the ASD with and without *SHANK3* deficiency.

In the present study, we employed SBM as well as SCN approaches to explore the disruptions of cortical surface morphology (i.e., both local features and anatomical networks) in ASD children with and without *SHANK3* deficiency. We hypothesized that there might be common as well as distinct disruptions of both local and networked cortical structural organization in ASD children with and without *SHANK3* deficiency, which may help to parse the heterogeneity of brain disturbances in ASD.

## Materials and Methods

### Participants

All participants were recruited at the Children’s Hospital of Fudan University [details about recruitment can be found in Liu et al. (2021)]. In total, 69 children in three groups [SHANK3 group, idiopathic ASD control, and typically developing (TD) control] were scanned and eight subjects were excluded for the analysis due to the poor quality of images. Children with recognizable lesions or abnormalities on scans, cerebral palsy, or other neurologic or degenerative diseases were excluded. SHANK3 group: 12 participants were enrolled with *SHANK3* deletion and/or mutation but without any other pathogenic or likely pathogenic variations [genetic testing details can be found in Liu et al. (2021)]. Idiopathic ASD control group: 24 ASD children without *SHANK3* deficiency or known genetic etiology were recruited. TD control group: 25 children underwent MRI scan because of the first episode of febrile convulsion and paroxysmal dizziness or headache, but otherwise normal developing children were used as normal controls.

Autism spectrum disorder diagnosis was based on the DSM-5 (Grzadzinski et al., 2013), ADOS-2 (Gotham et al., 2007), and Autism Diagnostic Interview-Revised (Lord et al., 1994) administrated by certified clinicians. Developmental and cognitive levels were evaluated by Griffiths Mental Development Scales (Griffiths; Benatti et al., 1986) conducted by licensed and certified clinicians.

### Image Acquisition and Preprocessing

T1-weighted images were acquired employing a high-resolution 3D T1-weighted BRAVO (BRA in Volume imaging) sequence with the GE 3.0 Tesla Discovery MR750 system (GE Medical Systems, Milwaukee, WI, United States) with a 32-channel head coil at the Radiology Department after parental consent. Sequence parameters were the following: repetition time (TR) = 8.2 ms; echo time (TE) = 3.2 ms; flip angle = 12°; voxel size = 1 mm × 1 mm × 1 mm; gap = 0; FOV = 256 mm × 256 mm; and matrix = 256 × 256. Participants were routinely sedated in the “Sedation Center” under the supervision of a licensed clinician or anesthesiologist. Children are given chloral hydrate at a dose of 50 mg/kg orally approximately 1 h prior to the MRI scan. Vital signs were monitored and recorded during the scan.

The images were imported in the CAT12^1^ toolboxes implemented in the Statistical Parametric Mapping software package (SPM12)^2^ on the platform of Matlab (Version R2014b, MathWorks Inc., Natick, MA, United States). All the T1-weighted images were then segmented into gray matter, white matter, and cerebrospinal fluid and spatially normalized using the DARTEL algorithm (Ashburner, 2007). Gray matter-segmented images were submitted to the Check Sample Homogeneity by using the function embedded in CAT-12. Then, the segmented images were smoothed with a 6-mm full-width at half-maximum (FWHM) Gaussian kernel. At the end of this preprocessing, modulated, smoothed, normalized images were obtained for statistical analysis. Besides, the image quality rating (IQR) generated by CAT12 served as the parameter for imaging quality control.

This study was approved by the ethics committee of Children’s Hospital of Fudan University, and all of the procedures were in accordance with the Declaration of Helsinki.

### Surface-Based Morphometry Analysis

To characterize cortical morphological features, an SBM analysis was conducted using the CAT12 toolbox to estimate CT and the central surface of hemispheres based on the projection-based thickness (PBT) approach (Dahnke et al., 2013). The left and right CT maps and the additional surface parameters, including fractal dimension (FD), GI, and SD, were resampled to the HCP 32k surface mesh and smoothed with a 15-mm FWHM Gaussian kernel for the CT estimates and 20-mm FWHM for the rest parameters as suggested by CAT 12. CT is obtained by projecting the local maxima of white matter distance after tissue segmentation onto other gray matter voxels, which measures the distance between the inner and outer surfaces (Dahnke et al., 2013). FD is defined as the slope of a logarithmic plot of surface area versus a measure of the bandwidth of frequencies in the reconstructed surface shape based on a spherical harmonic reconstruction approach, measuring the surface complexity (Yotter et al., 2011). Local GI is defined as the estimations of “smoothed absolute mean curvature” within a spherical surface mesh, revealing the degree of cortical convolution (Luders et al., 2006). SD is defined as the square root-transformed Euclidean distance between the central surface (half way between the inner and outer surfaces) and its convex hull.

### Structural Covariance Networks Approach

**FIGURE 1:**
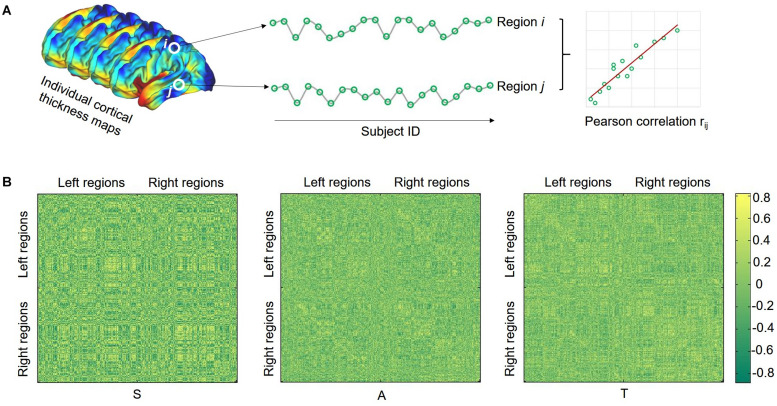
Illustration of calculating structural covariance connectivity between two regions **(A)** and structural covariance matrix for each group **(B)**. S, SHANK3 group; A, ASD group; and T, typical developing group.

Considering the popularity in previous studies, here we merely selected the CT for structural covariance estimation (He et al., 2007; Alexander-Bloch et al., 2013a). The effects of age, gender, and overall mean CT were regressed from the mean thickness of each brain region, and the residuals were used for the subsequent steps of the analysis. Structural covariance analysis was conducted in the regions of interest (ROIs) defined by the HCP-MMP1.0 atlas (Glasser et al., 2016) which contains 180 regions for each hemisphere. Pearson correlations were applied between the CT of each possible pair of ROIs to generate a 360 × 360 cortical covariance matrix for each group (Figure 1). Considering that the network organization of the human brain is economic and small-world, the covariance matrix was then binarized and sparsified (i.e., 15%) according to previous studies (Bassett et al., 2008; Yin et al., 2019). The graph theory was finally used to characterize the topological properties of the SCNs. Here, we employed a network efficiency measure to quantify the small-world property of the SCNs following our previous work (Yin et al., 2014). This efficiency metric can deal with disconnected graphs and provides a clear physical meaning for the topological characterization of the networks (Latora and Marchiori, 2001). The global efficiency (GE) of graph *G* can be calculated as


$$\text{GE}=\frac{1}{N\,\left(N-1\right)}\,\sum_{i\neq j\in G}\frac{1}{L_{i\,j}}$$

where *N* is the number of nodes; *L*_*ij*_ is the shortest path length between nodes *i* and *j* in graph *G* (i.e., the minimal number of edges that has to be traveled to go from nodes *i* to *j*).

The local efficiency (LE) of graph *G* is measured as


$$\text{LE}=\frac{1}{N}\,\sum_{i\in G}\text{GE}\,\left(G_{i}\right)$$

where *G*_*i*_ denotes the subgraph composed of the nearest neighbors of node *i*.

Practically, a network can be categorized as a small-world network if GE is slightly less than and LE is much greater than the matched random networks with the same number of nodes, edges, and degree distribution. For comparison purposes, we generated random networks using the random rewiring procedure that preserves degree distribution as the real network (Maslov and Sneppen, 2002). Thus, a small-world network should meet the following criteria: *LE (real)/LE (random) > 1* and *GE (real)/GE (random) ≈ 1* (Wang et al., 2009). To explore small-world efficiency, we also calculated the normalized *GE = GE (real)/GE (random)* and normalized *LE = LE (real)/LE (random)*.

In addition, we calculated the nodal degree, which is commonly used to identify network hubs. Degree is defined as the number of immediate neighbors of a given node.

### Statistical Analysis

Demographic and clinical data were analyzed using the statistical package SPSS 20.0. The threshold for all statistical significance was set at *p* < 0.05. Age, sex, developmental quotient, and total cranial volume were included as nuisance covariates.

For SBM, analysis of covariance (ANCOVA) was applied to the global mean values of each morphological parameter for the three groups followed by *post-hoc t*-tests. Age, sex, and total cranial volume were included as covariates. At the vertex-wise level, one-way ANOVA was conducted among the three groups and significant clusters with false discovery rate (FDR) corrected were further picked up in CAT12. For each metric, *post-hoc t*-tests between each two groups were performed taking the significant result of ANOVA as the explicit mask. Age, sex, and total intracranial volume were included as nuisance covariates. Significance was set at *p* < 0.05 (FDR corrected).

For SCNs, a nonparametric permutation test was carried out to identify statistical significance of the between-group differences in network metrics. Briefly, we first calculated the difference of each network metric between groups. A null distribution of the difference for each metric was then obtained by randomly reallocating each subject’s set of regional CT estimates to one or the other of the two groups and recomputing the difference of each metric between two randomized groups. Note that we calculated the network metrics for each randomized group using the same sparsity threshold as in the real brain networks. This randomization procedure was repeated 1,000 times, and the 95 percentile points of each distribution were used as the critical values for a one-tailed test of the null hypothesis with a probability of type I error of 0.05 (He et al., 2008). For the between-group comparisons of nodal degree, the FDR correction method was applied to correct multiple comparisons for *N* = 360 regions.

Spearman rank correlation analysis was conducted between the altered brain morphological features in SHANK3 and ASD groups and the clinical characteristics. Significance correction for multiple comparisons was applied using FDR correction (*p* < 0.05).

## Results

### Participant Characteristics

No significant differences in age (for the SHANK3 group, mean ± SD: 4.9 ± 3.2 years; for the ASD group, mean ± SD: 4.0 ± 1.9 years; and for the typically developed group, mean ± SD: 4.9 ± 2.4 years) and sex (for the SHANK3 group, 6 males and 6 females; for the ASD group, 20 males and 4 females; and for the typically developed group, 19 males and 6 females) between groups (all *p* values > 0.05). Compared to idiopathic ASD children, the SHANK3 group achieved similar scores in each domain and severity of ADOS-2 but exhibited a significantly worse performance in all of the developmental domains of Griffith (gross motor: *p* < 0.0001; social: *p* = 0.002; language: *p* < 0.0001; fine motor: *p* = 0.0004; adaptability: *p* = 0.006). Detailed demographic information and clinical characteristics by group are presented in Table 1.

**TABLE 1:** Summary of clinical characteristics of studying subjects.

	**SHANK3 group**	**ASD group**	**TD group**	***p* value**
Number of participants	12	24	25	
Gender, *n* (M/F)	6:6	20:4	19:6	0.095^*a*^
Age (*M* + *SD*)	4.9 ± 3.2	4.0 ± 1.9	4.9 ± 2.4	0.322^*b*^
ADOS-2 Scale (individuals)				
ASD severity	6.5 ± 1.67	6.68 ± 1.28	NA	0.570
SA	16.3 ± 3.86	15.59 ± 4.04	NA	0.720
RRB	1.58 ± 1.50	1.63 ± 1.17	NA	0.702
ADOS total score	17.92 ± 5.03	17.23 ± 4.46	NA	0.874
Griffith Scale (individuals)	11	20	NA	
Gross motor	50.3 ± 15.08	73.39 ± 16.87	NA	<0.0001^****^
Social	37.16 ± 17.22	60.20 ± 17.25	NA	0.002^**^
Language	17.79 ± 9.11	45.81 ± 19.35	NA	<0.0001^b^^****^
Fine motor	33.09 ± 18.65	57.71 ± 16.12	NA	0.0004^***^
Performance	33.34 ± 25.80	58.16 ± 22.47	NA	0.006^**^

*Unpaired student’s *t*-test (two-tailed).*

*^*a*^χ^2^ test.*

*^*b*^one-way ANOVA.*

*^∗∗^*p* < 0.01, ^∗∗∗^*p* < 0.001, and ^∗∗∗∗^*p* < 0.0001.*

*ADOS-2, Autism Diagnostic Observation Schedule-The Second Edition; SA, social affect; RRB, restricted and repetitive behavior; and NA, not applicable.*

### Inter-Group Differences in Surface-Based Morphometric Features

No group differences were found in all of the imaging quality control parameters, including the six absolute head motion parameters and IQR (for the SHANK3 group, mean ± SD: 0.907 ± 0.006; for the ASD group, mean ± SD: 0.906 ± 0.005; and for the typically developed group, mean ± SD: 0.908 ± 0.006; *p* > 0.05).

**FIGURE 2:**
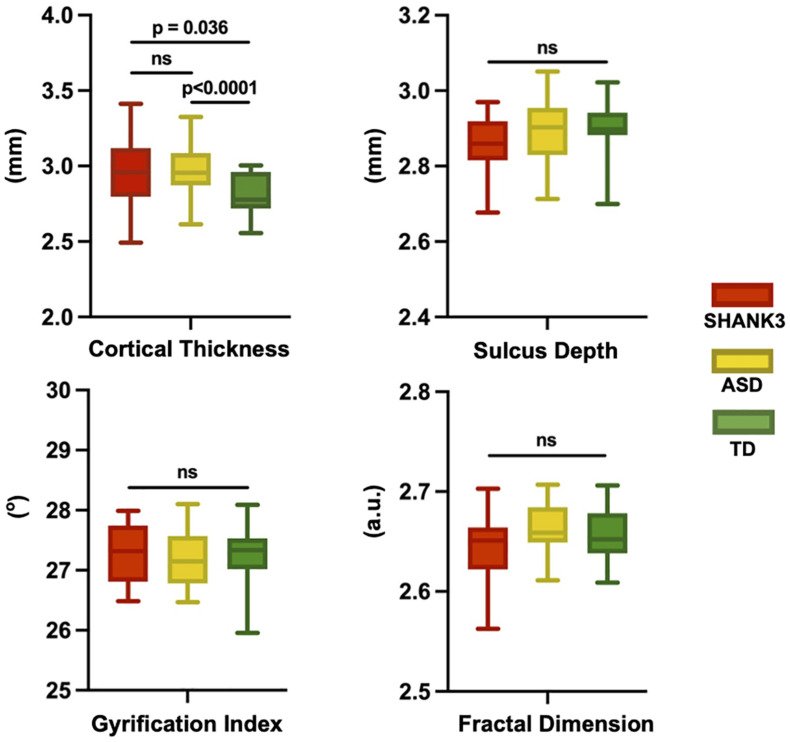
Global leveled ANCOVA analysis showed significant difference of cortical thickness among the three groups. *Post-hoc t*-tests revealed significantly decreased cortical thickness in the ASD group but increased cortical thickness in the SHANK3 group compared to the TD group. No significant differences were found for the fractal dimension and sulcus depth among the three groups for ANCOVA analysis. ns, not significant; a.u., arbitrary unit.

At the global level, we found that the averaged CT is 2.96 ± 0.57 mm for the SHANK3 group, 2.58 ± 0.55 mm for the ASD group, and 2.81 ± 0.5 mm for the TD group; the averaged FD in the SHANK3 group was 2.64 ± 0.34, in the ASD group 2.66 ± 0.35, and in the TD group 2.65 ± 0.35; the average SD in the SHANK3 group was 2.86 ± 0.77 mm, in the ASD group 2.89 ± 0.85 mm, and in the TD group 2.90 ± 0.85 mm; the average GI in the SHANK3 group was 27.29 ± 0.5°, in the ASD group 27.19 ± 0.45°, and in the TD group 27.28 ± 0.46°. For the individual raw values, please see Supplementary Table 1. ANCOVA analysis revealed a significant difference of CT among the three groups (*F* = 8.64, *p* = 0.001). In the *post-hoc* analysis, the ASD group showed significantly decreased CT than the TD group (*t* = 3.86, *p* < 0.0001) and the SHANK3 group showed increased CT than the TD group (*t* = 2.18, *p* = 0.036; Figure 2). No significant difference was found between the SHANK3 group and the ASD group for CT. In addition, no significant differences were found for the FD and SD among the three groups with ANCOVA analysis (Figure 2).

**FIGURE 3:**
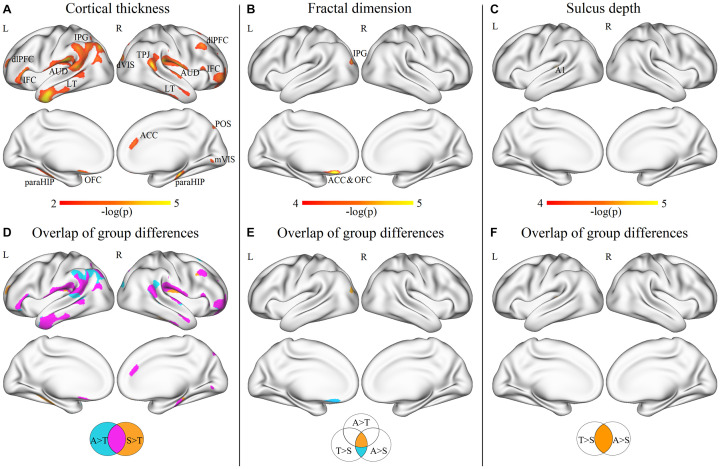
Vertex-wise ANOVA results and the overlap areas of intergroup differences. Significant differences of cortical thickness **(A)**, fractal dimension **(B)**, and sulcus depth **(C)** among ASD, SHANK3, and TD groups; the overlap areas of intergroup differences in cortical thickness **(D)**, fractal dimension **(E)**, and sulcus depth **(F)**. dlPFC, dorsolateral prefrontal cortex; IFC, inferior frontal cortex; AUD, auditory areas; LT, lateral temporal cortex; IPG, inferior parietal gyrus; dVIS, dorsal visual streams; TPJ, temporo-parieto-occipital junction; paraHIP, parahippocampus; OFC, orbitofrontal cortex; ACC, anterior cingulate cortex; mVIS, medial visual areas; POS, parieto-occipital sulcus; and A1, primary auditory cortex. A, ASD; T, TD; and S, SHANK3.

At the vertex level, through the vertex-wise ANOVA analysis of CT, we found significant differences among the three groups mainly in the regions of the dorsolateral prefrontal cortex, inferior frontal cortex (IFC), auditory areas, lateral temporal cortex (LT), inferior parietal gyrus, dorsal visual streams, temporo-parieto-occipital junction (TPJ), parahippocampus (paraHIP), orbitofrontal cortex (OFC), anterior cingulate cortex (ACC), medial visual areas, and parieto-occipital sulcus (*p* < 0.05, FDR corrected; Figure 3A). In the *post-hoc* analysis, the SHANK3 and ASD groups showed significantly increased CT mainly in the lateral temporal, prefrontal, primary auditory (part of superior temporal gyrus, STG), and temporo-parietal junction (TPJ) as well as limbic/paralimbic regions including the ACC, OFC, temporal pole, and paraHIP, compared with TD controls (*p* < 0.05, FDR corrected; Figure 3D). Notably, the SHANK3 group showed more disruptions in STG than the ASD group, while the latter showed more disruptions in the TPJ and inferior parietal lobule (IPL; Figure 3D). No significant difference was found between the SHANK3 group and the ASD group for CT. For the FD, ANOVA analysis revealed significant differences among the three groups mainly in the regions of the IPL, ACC, and OFC (Figure 3B). In the *post-hoc* analysis, the ASD group showed increased FD in a small region of the left IPL when compared with the SHANK3 and TD groups, while the SHANK3 group showed decreased FD in the left OFC in comparison with ASD and TD groups (Figure 3E). For the SD, the SHANK3 group showed decreased SD in the left primary auditory region (part of STG; Figures 3C,F) when compared with the ASD and TD groups. No group differences were found in local GI.

### Inter-Group Differences in Topological Properties of Structural Covariance Networks

**FIGURE 4:**
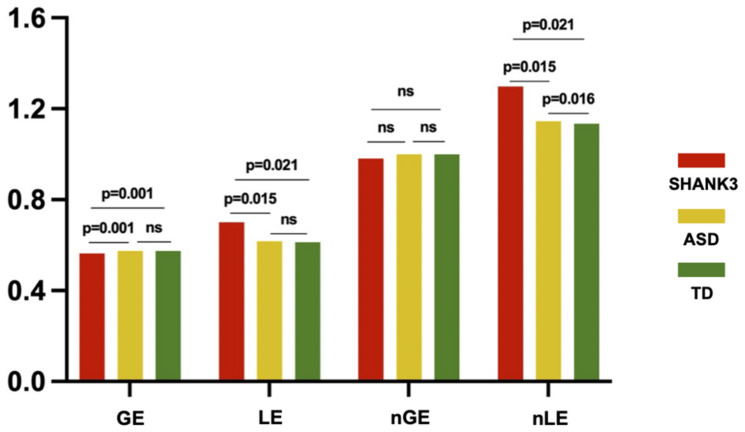
Intergroup differences in global efficiency and local efficiency between the SHANK3, ASD, and TD groups. The orange color indicates the SHANK3 group, the yellow color indicates the ASD group, and the green color indicates the TD group; network sparsity = 15%. ns, not significant; GE, global efficiency; LE, local efficiency; nGE, normalized global efficiency; and nLE, normalized local efficiency.

At the global level, we found that the *SHANK3*-deficient children exhibited a decrease in GE (*p* = 0.001) and an increase in LE (*p* = 0.021) compared to TD controls, while ASD children showed no significant differences in either global or LE compared with TD (*p* > 0.05). Compared with ASD children, *SHANK3*-deficient children exhibited a decrease in GE (*p* = 0.001) and an increase in LE (*p* = 0.015). In contrast, we found a significant increase in normalized LE in *SHANK3*-deficient compared with ASD groups, as well as in ASD compared with TD groups, while no significant differences were observed in normalized GE among the three groups (Figure 4).

**FIGURE 5:**
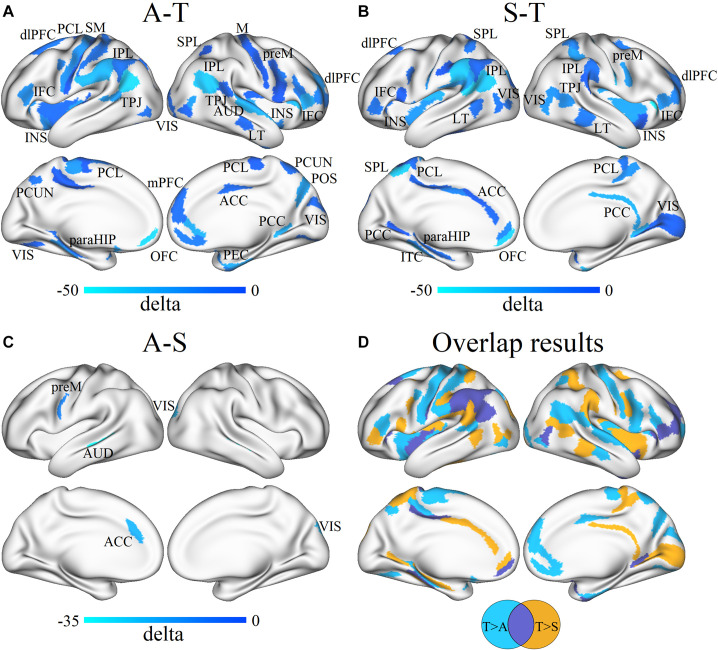
Differences in the group mean of nodal degree between ASD, SHANK3, and TD groups and the overlap results. Group differences in nodal degree between ASD and TD **(A)**, SHANK3 and TD **(B)**, and ASD and SHANK3 **(C)**, and overlap results between the group differences **(D)**. Blue and light blue colors indicate ASD < TD in **(A)**, SHANK3 < TD in **(B)**, and ASD < SHANK3 in **(C)**, respectively. dlPFC, dorsolateral prefrontal cortex; PCL, paracentral lobule; SM, somatosensory and motor cortex; IFC, inferior frontal cortex; INS, insular; IPL, inferior parietal lobule; TPJ, temporo-parieto-occipital junction; VIS, visual areas; SPL, superior parietal lobule; M, motor cortex; preM, premotor cortex; AUD, auditory areas; LT, lateral temporal cortex; ACC, anterior cingulate cortex; PCC, posterior cingulate cortex; PCUN, precuneus; POS, parieto-occipital sulcus; paraHIP, parahippocampus; OFC, orbitofrontal cortex; and PEC, perirhinal and ectorhinal_cortex. A, ASD; T, TD; and S, SHANK3.

For regional network analysis, group differences in nodal degree between every two groups were detected (all *p* < 0.05, FDR corrected). In contrast with TD controls, ASD without *SHANK3* deficiency showed significantly decreased nodal degrees mainly in sensorimotor areas, the left insular, and the right ACC and dorsomedial PFC (Figure 5A), while ASD with *SHANK3* deficiency showed decreased nodal degrees mainly in the left ACC, and the right insular cortex, posterior cingulate cortex (PCC), and medial visual cortex (Figure 5B). To overlap the between-group results, we observed a common reduction of the nodal degree in the prefrontal, IPL, and insular areas in both ASD with and without *SHANK3* deficiency compared with TD controls (Figure 5D). To compare with the ASD group, the SHANK3 group showed an increment of nodal degree in the ACC, premotor cortex, primary auditory cortex (part of STG), and visual areas in contrast with the ASD group (Figure 5C). Notably, the regions of disruptions of nodal degree in these two groups were geometrically close but distinct in the paracentral lobule, IFC, LT, and superior and IPL (Figure 5D).

### Correlations Between Clinical Characteristics and Cortical Morphology

**FIGURE 6:**
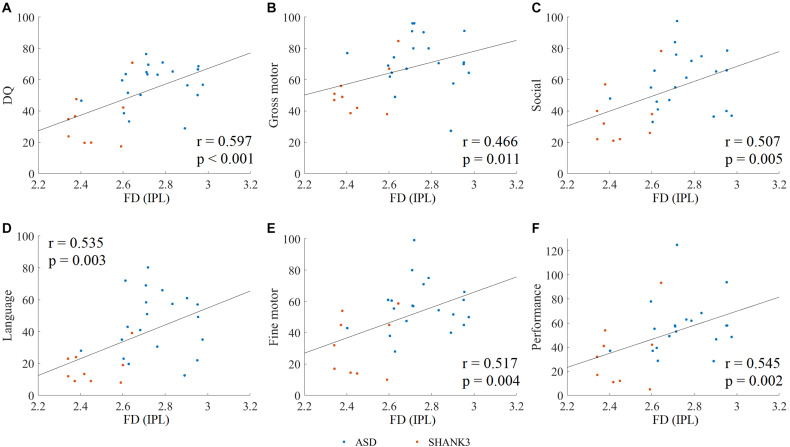
Spearman rank correlation between clinical assessments and altered fractal dimension (FD) in ASD and SHANK3 groups. The Spearman rank correlation between the altered FD and DQ scores **(A)**, gross motor **(B)**, social **(C)**, language **(D)**, fine motor **(E)**, and performance **(F)**. IPL, inferior parietal lobule. A, ASD; S, SHANK3; and T, TD.

Spearman rank correlation analysis was conducted to explore the relationship between the disruptions of cortical morphological features and clinical assessments in ASD and SHANK3 groups, and then FDR correction was performed (corrected *p* < 0.05). The disruptions of FD in a region of the left IPL in both ASD children with and without *SHANK3* deficiencies were related to general DQ (*r* = 0.597, *p* < 0.001), gross motor (*r* = 0.466, *p* = 0.011), social (*r* = 0.507, *p* = 0.005), language (*r* = 0.535, *p* = 0.003), fine motor (*r* = 0.517, *p* = 0.004), and performance (*r* = 0.545, *p* = 0.002; Figure 6). Correlations between other cortical morphological features (incl. CT, SD, and GI) and the clinical assessments were not significant.

## Discussion

Our study demonstrated some common and distinct disruptions of both local and networked cortical structural organization in ASD children with and without *SHANK3* defects. Locally, increased CT was found in both ASD children with and without *SHANK3* defects mainly in the LT, PFC, TPJ, STG, and limbic/paralimbic regions. As to the networked cortical organizations, the majority of the above regions were observed with reduction of the nodal degree. Fitting with the idea that *SHANK3* deficiency shares some common patterns of cortical structural alterations with ASD, *SHANK3* deficiency thus provides a unique opportunity to understand the underlying neuropathological mechanisms as a homogenous subgroup of ASD.

The social deficits and repetitive and stereotyped behaviors are the core symptoms of ASD (Grzadzinski et al., 2013). It is well known that language expression is the vital part of social interaction. The clinical manifestations of *SHANK3* deficiency include ASD, global developmental delay, delayed or absent speech, intellectual disability, and motor abnormalities (Soorya et al., 2013; Leblond et al., 2014). The penetrance of the ASD phenotype in patients with *SHANK3* deficiency has been reported from 84.4 to 92.8% (Soorya et al., 2013; Liu et al., 2021). In consistency with these previous reports, our current clinical assessment data demonstrated that both ASD children with and without *SHANK3* defects showed social interaction impairments and repetitive behaviors (ADOS-2) as well as language developmental delay (Griffith language domain). Along with the clinical data, increased CT as well as networked alterations were revealed in the PFC, TPJ, STG, and limbic/paralimbic regions. The PFC, TPJ, and STG are the most consistently reported neural correlates of social-cognitive functions (Wolf et al., 2018).

Superior temporal gyrus, responsible for language comprehension, face processing, and social cognition, has been proved to be associated with ASD (Bigler et al., 2007; Jou et al., 2010; Ameis and Catani, 2015). As the area where the temporal and parietal lobes meet, the TPJ incorporated information from the thalamus, the limbic system, and somatosensory systems and plays an important role in social perception as well as theory of mind (ToM; Poletti et al., 2012; Bitsch et al., 2018). Neuropsychological studies suggested that individuals with ASD showed ToM deficits which result in the disabilities to initiate and sustain reciprocal social interactions (DiLalla et al., 2017; Tordjman et al., 2019). The PFC, even in cross-species ASD models, has been proved to be the critical hub for emotional and social behavior, as well as executive functions (Chini and Hanganu-Opatz, 2021; Yan and Rein, 2021). Additionally, the limbic/paralimbic structural alterations in ASD suggested that the paralimbic–limbic system is closely related to repetitive and stereotyped behaviors (Catani et al., 2013; Li et al., 2018).

From the perspective of the brain network, we found that the SHANK3 group showed a significant increase in LE compared with ASD and TD groups. Instead, the GE in the SHANK3 group is slightly lower than that in ASD and TD groups. No significant differences in either local or GE were observed between ASD and TD groups. This result suggests that the configuration of the SCN in *SHANK3* children shifts toward a topology of regular network, which is a nonoptimal network configuration. Actually, the trend toward a regular network topology has often been observed in other neuropsychiatric disorders (De Vico Fallani et al., 2007; Yin et al., 2014). In contrast, the ASD children without *SHANK3* defects appear to reserve a relatively intact global topology in SCN.

At the nodal level, our results revealed a common reduction of the nodal degree as the networked parameter alterations in the PFC, IPL, and insular cortex in both ASD children with and without *SHANK3* defects. These findings were consistent with our previous assumptions that there might be possible alterations in large-scale brain networks in ASD children with and without *SHANK3* defects (Liu et al., 2021). IPL and dorsolateral PFC are the core components of the frontoparietal network (FPN) or also known as central executive network (CEN) which is crucial for attention sustenance, decision-making, working memory, and cognitive control (Seeley et al., 2007; van Oort et al., 2017). FPN/CEN is one of the three networks in the “triple-network model,” along with the salience network (SN) and the default mode network (DMN; van Oort et al., 2017; Wei et al., 2019). Disruption of FPN may account for the impairment in cognitive flexibility in ASD (Holiga et al., 2019), and reshaping of FPN connectivity might improve the cognitive–behavioral problems in ASD (De Luca et al., 2021). TPJ and PFC are part of DMN which is implicated in self-monitoring, ToM, and social cognition (Buckner et al., 2008; Sarasua et al., 2014). Functional connectivity studies have provided evidence for abnormal connectivity pattern of DMN and its subnetworks in individuals with ASD (von dem Hagen et al., 2013; Abbott et al., 2016). SN, which is composed of insula and ACC, orients attention toward salient information and facilitates switching between the FPN and DMN (Hermans et al., 2014; van Oort et al., 2020). Children with ASD exhibited both functional “over-” and “under-” connectivities in between the SN, DMN, and CEN (Lawrence et al., 2020; van Oort et al., 2020). Our morphological and network-based structural disruptions are consistent with these prior studies which may reflect a structural basis for the functional imbalance between these large-scale brain networks.

Interestingly, besides the common alterations, our study revealed some distinct abnormalities in ASD children with *SHANK3* defects compared to those without. Locally, more changes in STG and OFC were exhibited in ASD children with *SHANK3* defects, while those without *SHANK3* defects showed more changes in TPJ and IPL. On the networked level, ASD children with *SHANK3* defects showed more alterations of nodal degrees in ACC and PCC and right insula, while ASD without *SHANK3* defects exhibited more disruptions in the sensorimotor areas, the left insula, and dorsomedial PFC. SHANK3 protein is expressed highest in the cortico-striatal glutamatergic synapses (Shepherd, 2013; Monteiro and Feng, 2017). The medial orbitofrontal cortico-striatal loop originates in the OFC then projects to the caudate, putamen, and pallidum and then closes with projections returning to the OFC which shares reciprocal connections with ACC, PCC, STG, hippocampus, and parahippocampal cortex (Elliott et al., 2000; Jarbo and Verstynen, 2015). The current results supported the assumption of our previous study which suggested dysfunction of cortico-striatal connectivity in children with *SHANK3* defects (Liu et al., 2021).

Although large-scale studies sequenced numerous patient samples, there was only a maximum of 30% of ASD cases identified with genetic causes with no single gene contributing to greater than 2% of cases (Boyle et al., 2017; Dias and Walsh, 2020). One of the major reasons for the explanation is generally acknowledged as the etiological and clinical heterogeneity of ASD. Our monogenetic subgroup of ASD children with *SHANK3* defects exhibited both common and distinct disruptions in local and networked structural organization compared to those idiopathic ASD, suggesting both common and distinct neuropathological mechanisms underlying ASD with and without *SHANK3* deficiency. Differentiating the heterogeneity in ASD is critical to both clinicians and researchers to understand the phenotype and pathophysiology of the syndrome (Agelink van Rentergem et al., 2021; Ziats et al., 2021). Our study of monogenic ASD patients may thus offer new insights into the diverse findings of previous neuroimaging studies of idiopathic ASD.

There were several limitations of the current study. Although the sample size of the SHANK3 group used in the current study is relatively large compared with previous studies (Monteiro and Feng, 2017), it is still small due to the rare condition of *SHANK3* mutations. In particular, even though the PBT method is better than other approaches on the basis of root mean square error, it still represents a thickness measurement error of 0.39 ± 0.02 mm (Dahnke et al., 2013). This may potentially confound the observed between-group differences in CT. Future studies with expanded sample size need to verify the current results. Besides, the current MRI scans were mainly structural so that future studies of functional connectivity may allow for more in-depth understanding of our conclusions. Despite these limitations, our results provide some new guidance to further understand the symptoms presented by ASD children both with and without *SHANK3* defects.

## Conclusion

Besides commonality, our findings have revealed dissociable patterns of alterations in both local and networked features of brain morphology between ASD children with and without *SHANK3* defects, which suggests new insight into the understanding of neuropathological mechanisms underlying ASD. Moreover, this monogenic study may provide a valuable path for parsing the heterogeneity of brain disturbances in ASD.

## Data Availability Statement

The datasets presented in this study can be found in online repositories. The names of the repository/repositories and accession number(s) can be found in the article/Supplementary Material.

## Ethics Statement

The studies involving human participants were reviewed and approved by Ethics Committee of Children’s Hospital of Fudan University. Written informed consent to participate in this study was provided by the participants’ legal guardian/next of kin.

## Author Contributions

DL analyzed and interpreted the data and wrote and reviewed the manuscript. XX designed the research, interpreted the data, and reviewed the manuscript. DY analyzed and interpreted the data and reviewed the manuscript. ZQ interpreted the data and reviewed the manuscript. CL collected and analyzed the clinical data. ZH analyzed and interpreted the imaging data. HL, QX, BZ, CH, YZ, and YW prepared the data and reviewed the manuscript. JN reviewed the manuscript. All authors contributed to the article and approved the submitted version.

## Conflict of Interest

The authors declare that the research was conducted in the absence of any commercial or financial relationships that could be construed as a potential conflict of interest.

## Publisher’s Note

All claims expressed in this article are solely those of the authors and do not necessarily represent those of their affiliated organizations, or those of the publisher, the editors and the reviewers. Any product that may be evaluated in this article, or claim that may be made by its manufacturer, is not guaranteed or endorsed by the publisher.

## Footnotes

1
http://dbm.neuro.uni-jena.de/cat/

2
http://www.fil.ion.ucl.ac.uk/spm/software/spm12/
