# Supplementary material for: Common and Distinct Disruptions of Cortical Surface Morphology Between Autism Spectrum Disorder Children With and Without SHANK3 Deficiency
Source: Front Neurosci. 2021 Oct 28;15:751364. doi: 10.3389/fnins.2021.751364 (PMC8581670; doi:10.3389/fnins.2021.751364)
Supplement: Supplementary file 1 [file Table_1.docx]

**Common and distinct disruptions of cortical surface morphology between ASD children with and without *SHANK3* deficiency**

**Dongyun Li^1^, Chunxue Liu^1^, Ziyi Huang^3,4^, Huiping Li^1^, Qiong Xu^1^, Bingrui Zhou^1^, Chunchun Hu^1^, Ying Zhang^1^, Yi Wang^1^, Jingxin Nie^4^, Zhongwei Qiao^2*^, Dazhi Yin^3*^, Xiu Xu^1*^**

^1^Department of Child Health Care, Children's Hospital of Fudan University, 399 Wanyuan Road, Shanghai 201102, China

^2^ Department of Radiology, Children's Hospital of Fudan University, 399 Wanyuan Road, Shanghai 201102, China

^3^Shanghai Key Laboratory of Brain Functional Genomics (Ministry of Education), School of Psychology and Cognitive Science, Affiliated Mental Health Center,East China Normal University, Shanghai 200062, China

^4^School of Psychology, South China Normal University, Guangzhou 510631, China

**^*^Correspondence**:

Xiu Xu

xuxiu@shmu.edu.cn

Dazhi Yin

dzyin@psy.ecnu.edu.cn

Zhongwei Qiao

qiaozhwei@163.com

This file contains Supplementary Table 1.

**Supplementary Table 1. Cortical thickness, fractal dimension and sulcus depth for each subject in three groups at the global level**

|  | **ASD group** | **SHANK3 group** | **TD group** |
| --- | --- | --- | --- |
| **Cortical Thickness** | 2.874364 | 3.021373 | 2.616106 |
|  | 2.920221 | 2.961448 | 2.777897 |
|  | 2.992949 | 2.957587 | 2.956515 |
|  | 2.860256 | 3.131968 | 3.004893 |
|  | 2.895617 | 2.903064 | 2.758864 |
|  | 3.069458 | 2.491786 | 2.632024 |
|  | 2.874735 | 2.753038 | 2.716418 |
|  | 2.86973 | 3.114294 | 2.72281 |
|  | 2.613621 | 3.412812 | 2.735564 |
|  | 3.273317 | 2.81271 | 3.002523 |
|  | 2.851942 |  | 2.572395 |
|  | 3.113266 |  | 2.755604 |
|  | 3.079989 |  | 2.889106 |
|  | 3.024811 |  | 2.970547 |
|  | 2.889256 |  | 2.71671 |
|  | 2.815454 |  | 2.985387 |
|  | 3.066618 |  | 2.723117 |
|  | 3.076153 |  | 2.740391 |
|  | 2.911561 |  | 2.842896 |
|  | 3.326146 |  | 2.963796 |
|  | 3.105685 |  | 2.555567 |
|  | 3.103925 |  | 2.952988 |
|  |  |  | 2.982418 |
|  |  |  | 2.912623 |
|  |  |  | 2.799781 |
|  | **ASD group** | **SHANK3 group** | **TD group** |
| **Fractal Dimension** | 2.618431 | 2.662253 | 2.652107 |
|  | 2.669879 | 2.593774 | 2.680977 |
|  | 2.697828 | 2.646417 | 2.638942 |
|  | 2.658553 | 2.562615 | 2.637904 |
|  | 2.68526 | 2.655512 | 2.680095 |
|  | 2.658746 | 2.633254 | 2.627933 |
|  | 2.683549 | 2.667082 | 2.624677 |
|  | 2.611159 | 2.631666 | 2.608994 |
|  | 2.684144 | 2.663126 | 2.622979 |
|  | 2.656366 | 2.702985 | 2.638155 |
|  | 2.70473 |  | 2.669315 |
|  | 2.686773 |  | 2.615721 |
|  | 2.706787 |  | 2.706118 |
|  | 2.643535 |  | 2.647421 |
|  | 2.666952 |  | 2.658847 |
|  | 2.650778 |  | 2.652209 |
|  | 2.659144 |  | 2.640683 |
|  | 2.659019 |  | 2.677957 |
|  | 2.650053 |  | 2.639005 |
|  | 2.635445 |  | 2.678986 |
|  | 2.645663 |  | 2.659109 |
|  | 2.658609 |  | 2.695318 |
|  | 2.618431 |  | 2.662495 |
|  | 2.669879 |  | 2.659031 |
|  | 2.697828 |  | 2.699211 |
|  |  |  | 2.652107 |
|  |  |  | 2.680977 |
|  |  |  | 2.638942 |
|  | **ASD group** | **SHANK3 group** | **TD group** |
| **Sulcus Depth** | 2.893747 | 2.969459 | 2.893982 |
|  | 2.80986 | 2.916674 | 2.931106 |
|  | 2.886932 | 2.859875 | 2.868601 |
|  | 2.793905 | 2.860014 | 2.933038 |
|  | 3.00798 | 2.677207 | 2.943932 |
|  | 2.966697 | 2.856754 | 2.8977 |
|  | 2.773522 | 2.927149 | 2.835217 |
|  | 2.83648 | 2.80751 | 2.888563 |
|  | 2.950201 | 2.818282 | 2.947051 |
|  | 2.759956 | 2.863158 | 2.699368 |
|  | 2.93838 |  | 2.942275 |
|  | 2.903839 |  | 2.896585 |
|  | 3.050899 |  | 3.022046 |
|  | 2.901855 |  | 2.929284 |
|  | 2.937513 |  | 2.875832 |
|  | 2.974722 |  | 2.891907 |
|  | 2.865057 |  | 2.934839 |
|  | 2.976362 |  | 2.87627 |
|  | 2.922631 |  | 2.887797 |
|  | 2.712803 |  | 2.95358 |
|  | 2.93819 |  | 2.993831 |
|  | 2.888019 |  | 2.900282 |
|  | 2.893747 |  | 2.708428 |
|  | 2.80986 |  | 2.940625 |
|  | 2.886932 |  | 2.895837 |
|  |  |  | 2.893982 |
|  |  |  | 2.931106 |
|  |  |  | 2.868601 |
|  | **ASD group** | **SHANK3 group** | **TD group** |
| **Gyrification Index** | 27.18911 | 27.42829 | 27.74069 |
|  | 27.22021 | 27.68902 | 27.4277 |
|  | 26.90484 | 27.98822 | 26.99531 |
|  | 26.80247 | 27.33091 | 27.35674 |
|  | 27.5349 | 26.85375 | 27.55451 |
|  | 26.71156 | 27.91592 | 27.45802 |
|  | 27.10759 | 26.67462 | 27.41252 |
|  | 28.10205 | 27.25098 | 27.82589 |
|  | 27.46136 | 27.30248 | 27.49965 |
|  | 26.4707 | 26.48485 | 27.05582 |
|  | 27.6701 |  | 27.54906 |
|  | 27.07387 |  | 28.08823 |
|  | 27.04598 |  | 25.95684 |
|  | 26.63177 |  | 27.31885 |
|  | 27.70661 |  | 27.2675 |
|  | 26.66476 |  | 27.33425 |
|  | 27.6773 |  | 27.5162 |
|  | 26.63523 |  | 27.27443 |
|  | 27.35386 |  | 27.32721 |
|  | 26.99404 |  | 27.0438 |
|  | 27.41955 |  | 26.97698 |
|  | 27.93319 |  | 26.64175 |
|  | 27.18911 |  | 28.00896 |
|  | 27.22021 |  | 26.95372 |
|  | 26.90484 |  | 26.53767 |
|  |  |  | 27.74069 |
|  |  |  | 27.4277 |
|  |  |  | 26.99531 |
